# Emergency treatment with levetiracetam or phenytoin in status epilepticus in children—the EcLiPSE study: study protocol for a randomised controlled trial

**DOI:** 10.1186/s13063-017-2010-8

**Published:** 2017-06-19

**Authors:** Mark D. Lyttle, Carrol Gamble, Shrouk Messahel, Helen Hickey, Anand Iyer, Kerry Woolfall, Amy Humphreys, Naomi E. A. Bacon, Louise Roper, Franz E. Babl, Stuart R. Dalziel, Mary Ryan, Richard E. Appleton, supported by Paediatric Emergency Research in the United Kingdom & Ireland (PERUKI)

**Affiliations:** 1 Bristol Royal Hospital for Children, Bristol, UK; 2 Faculty of Health and Applied Sciences, University of the West of England, Bristol, UK; 3 Department of Biostatistics, University of Liverpool, Liverpool, UK; 4 Alder Hey Children’s Health Park, Eaton Road, Liverpool, L12 2AP UK; 5 Medicines for Children Clinical Trials Unit, University of Liverpool, Institute of Child Health, Alder Hey Children’s NHS Foundation Trust, Liverpool, UK; 6 Institute of Psychology, Health and Society, University of Liverpool, Liverpool, UK; 7 Royal Children’s Hospital, Melbourne, Australia; 8 Murdoch Children’s Research Institute, Melbourne, Australia; 9 Faculty of Medicine, Dentistry and Health Sciences, University of Melbourne, Melbourne, Australia; 10 Starship Hospital, Auckland, New Zealand; 11 Liggins Institute, University of Auckland, Auckland, New Zealand

**Keywords:** Status epilepticus, Paediatric, Seizure, Levetiracetam, Phenytoin, Randomised, Trial

## Abstract

**Background:**

Convulsive status epilepticus (CSE) is the most common life-threatening neurological emergency in childhood. These children are also at risk of significant morbidity, with acute and chronic impact on the family and the health and social care systems. The current recommended first-choice, second-line treatment in children aged 6 months and above is intravenous phenytoin (fosphenytoin in the USA), although there is a lack of evidence for its use and it is associated with significant side effects. Emerging evidence suggests that intravenous levetiracetam may be effective as a second-line agent for CSE, and fewer adverse effects have been described. This trial therefore aims to determine whether intravenous phenytoin or levetiracetam is more effective, and safer, in treating childhood CSE.

**Methods/design:**

This is a phase IV, multi-centre, parallel group, randomised controlled, open-label trial. Following treatment for CSE with first-line treatment, children with ongoing seizures are randomised to receive either phenytoin (20 mg/kg, maximum 2 g) or levetiracetam (40 mg/kg, maximum 2.5 g) intravenously. The primary outcome measure is the cessation of all visible signs of CSE as determined by the treating clinician. Secondary outcome measures include the need for further anti-seizure medications or rapid sequence induction for ongoing CSE, admission to critical care areas, and serious adverse reactions. Patients are recruited without prior consent, with deferred consent sought at an appropriate time for the family. The primary analysis will be by intention-to-treat. The primary outcome is a time to event outcome and a sample size of 140 participants in each group will have 80% power to detect an increase in CSE cessation rates from 60% to 75%. Our total sample size of 308 randomised and treated participants will allow for 10% loss to follow-up.

**Discussion:**

This clinical trial will determine whether phenytoin or levetiracetam is more effective as an intravenous second-line agent for CSE, and provide evidence for management recommendations. In addition, this trial will also provide data on which of these therapies is safer in this setting.

**Trial registration:**

ISRCTN identifier, ISRCTN22567894. Registered on 27 August 2015

EudraCT identifier, 2014-002188-13. Registered on 21 May 2014

NIHR HTA Grant: 12/127/134

**Electronic supplementary material:**

The online version of this article (doi:10.1186/s13063-017-2010-8) contains supplementary material, which is available to authorized users.

## Background

Convulsive status epilepticus (CSE) is the most common life-threatening neurological emergency in children, with an incidence of 20 per 100,000 children per year [1, 2]. It is the second most common reason for unplanned admission to paediatric intensive care units (PICUs) in the UK, accounting for 5.6% of all PICU admissions [3]. These children are also at increased risk of irreversible morbidity including chronic drug-resistant epilepsy, neurodisability, and learning difficulties, which result in major long-term demands on acute and chronic health and social care resources [4].

The current UK emergency care pathway for the management of childhood CSE is the step-wise algorithm advocated in advanced paediatric life support (APLS) [5]. First-line treatment is two doses of a benzodiazepine given 10 min apart; if the child continues to fit 10 min after the second dose of benzodiazepine, a second-line anticonvulsant is administered. APLS recommends phenytoin as the first-choice second-line anticonvulsant; if the child is allergic to phenytoin, has previously not responded to it, or has experienced a serious adverse event (SAE), phenobarbital is recommended. Failure to stop CSE necessitates rapid sequence induction (RSI), intubation, and admission to the PICU, with consequent potential for iatrogenic consequences including pneumonia, hospital-acquired infections, and prolonged admission.

There is an absence of randomised evidence to support the use of phenytoin as the second-line anticonvulsant despite its use as a standard intravenous (IV) anticonvulsant for the treatment of CSE since the 1940s. A retrospective case note review in which 87% (331/381) children administered a second-line anticonvulsant received phenytoin reported seizure cessation in 190 cases (50%) [6]. There is considerably more literature on phenytoin’s potential adverse effects, including potentially fatal cardiac arrhythmias and Stevens-Johnson syndrome (Table 1) [7–9]. The risk of a cardiac arrhythmia is related to the rate of infusion and phenytoin is therefore infused over at least 20 min.

**Table 1:** Comparison of phenytoin and levetiracetam

	Phenytoin	Levetiracetam
Easy to administer^a^	☒	☑
Rapid onset of action^a^	☑	☑
Intermediate to long action^a^	☑	☑
Broad spectrum^a^	☒	☑
Minimal morbidity^a^	☒	☑
Useful as maintenance AED^a^	☑	☑
IV solution compatibility^a^	☒	☑
Rate of infusion	20 min (minimum)	5 min (minimum)
Effectiveness in CSE (seizure termination rate)	50–60%	75–100%
Evidence base		
Cost		
Interactions with other drugs		
Adverse effects		
Potentially fatal	Cardiac arrhythmia Cardiac asystole Stevens-Johnson syndrome	
Non-fatal	Hypotension Hepatotoxicity Phlebitis Severe extravasation injury (‘purple glove syndrome’)	Dizziness Somnolence Headache Agitation IV site irritation

^a^Characteristics of ideal intravenous AEDs as outlined by Wheless and Treiman [41]

*AED* anti-epileptic drug, *CSE* convulsive status epilepticus, *IV* intravenous

Levetiracetam is a broad-spectrum anticonvulsant which effectively treats focal and generalised tonic-clonic and myoclonic seizures. A growing body of evidence, predominantly but not exclusively anecdotal, suggests that IV levetiracetam is safe and effective in the treatment of acute repetitive seizures and both convulsive and non-convulsive status epilepticus, with reported seizure cessation rates between 76 and 100% [10–18]. Both levetiracetam and lorazepam seem to be equally effective in terminating CSE (relative risk 0.97, 95% confidence interval 0.44 to 2.13) [19]. A systematic review published in 2012 indicated that efficacy ranged from 44 to 94% with reported higher rates in retrospective studies [20]. Reported IV levetiracetam doses range from 20 to 60 mg/kg with infrequent and mild adverse effects even at the upper extreme of the dose range [21]. These include dizziness, somnolence, headache, and transient agitation, but there have been no reports of arrhythmias, hypotension, tissue extravasation reactions, Stevens-Johnson syndrome, or hepatotoxicity (Table 1) [21, 22]. Levetiracetam can be infused over 5–7 min, which suggests that, theoretically, CSE may be terminated more rapidly than with phenytoin. Consequently, a reasonable hypothesis is that levetiracetam may be more effective and safer than intravenous phenytoin in terminating CSE [10, 23, 24].

CSE management has been identified as a key priority area for research by a number of sources including the national paediatric emergency medicine research network (Paediatric Emergency Research in the United Kingdom & Ireland; PERUKI) [25] in their inaugural prioritisation exercise [26], and the National Institute for Health and Care Excellence (NICE) in their update of their national epilepsy guideline published in January 2012 [27]. A high-quality randomised controlled trial (RCT) is therefore essential to determine whether phenytoin or levetiracetam is the ideal drug in CSE, as highlighted in the recent systematic review [20].

The EcLiPSE study (Emergency treatment with Levetiracetam or Phenytoin in Status Epilepticus in children) is a phase IV, multi-centre, parallel group, randomised controlled, open-label trial comparing IV levetiracetam with IV phenytoin. The study objectives are to determine which treatment is: 1) more effective as a second-line anticonvulsant for the management of childhood CSE; 2) associated with fewer adverse effects.

## Methods

The protocol for this study has been written in accordance with the Standard Protocol Items: Recommendations for Interventional Trials (SPIRIT) checklist (Additional file 1).

### Study setting

Thirty Emergency Departments (ED) throughout all regions of the UK are participating, selected from the membership of PERUKI, a collaborative paediatric emergency medicine research network [25]. Participating sites may be tertiary or district general hospitals with EDs that treat either children alone, or children and adults. A full list of participating centres is available on the EcLiPSE website (http://www.eclipse-study.org.uk/). Centres are selected based on factors including site research infrastructure, number of likely recruits, and proposed training strategy.

### Study population

#### Inclusion criteria

Children aged 6 months to <18 years who present with generalised tonic-clonic, generalised clonic, or focal clonic CSE that requires second-line treatment, provided that first-line treatment has been administered according to APLS guidelines [5] or the child’s personalised rescue care plan. If patients are given more than two doses of benzodiazepines (for example, in the community and then the ED), or fewer than two doses (for example, due to previous benzodiazepine sensitivity), or where the personalised care plan includes rectal paraldehyde as first-line treatment, then they are still eligible. Children receiving oral phenytoin or levetiracetam as maintenance therapy are eligible.

#### Exclusion criteria

Children are excluded if they: 1) present with absence, myoclonic, or non-convulsive status epilepticus, or infantile spasms; 2) are known or suspected to be pregnant; 3) have a known contraindication or allergy to levetiracetam or phenytoin; 4) have known established renal failure; 5) have been given a second-line antiepileptic drug during this episode of CSE prior to eligibility assessment; or 6) are known to have previously been treated in the EcLiPSE study.

### Outcome measures

The primary outcome is time from randomisation to cessation of all visible signs of CSE activity. The secondary outcomes are: 1) need for further anticonvulsants to manage seizures after randomised treatment; 2) need for RSI due to ongoing CSE; 3) need for admission to a PICU or high-dependency unit (HDU); and 5) serious adverse reactions (SARs) including death, airway complications, cardiovascular instability, extravasation injury, and extreme agitation.

### Screening, randomisation, recruitment, and consent

**Fig. 1:**
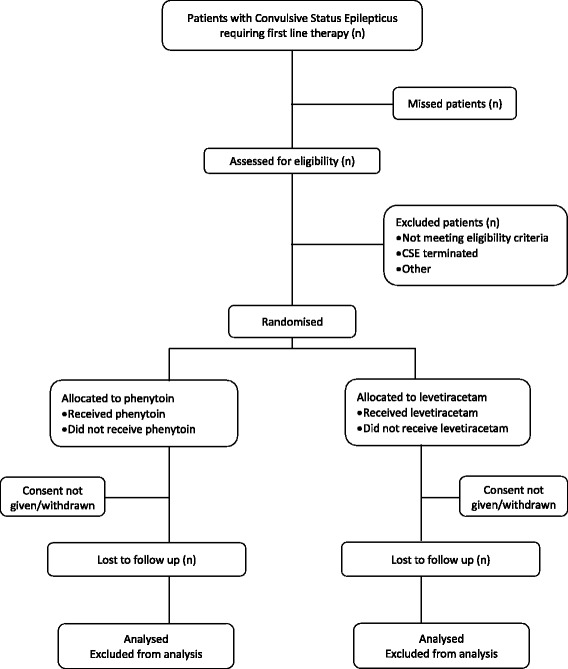
Emergency treatment with Levetiracetam or Phenytoin in Status Epilepticus in children (EcLiPSE) study flowchart. *CSE* convulsive status epilepticus

The overall study flowchart is presented in Fig. 1.

#### Screening

Screening commences once a child arrives in the ED and has received first-line treatment for CSE. A unique participant screening form is used which includes an eligibility assessment and reasons for non-randomisation where appropriate.

#### Randomisation and recruitment

Eligible children are randomised following completion of first-line therapy if the CSE is ongoing, enabling preparation and administration of the allocated treatment in a timeframe consistent with APLS guidance [5]. If the CSE terminates prior to administration of the allocated treatment then the patient may still be given that treatment if the CSE restarts while they remain in the ED. Randomised participants who do not receive a second-line treatment in the ED will not be included in the primary outcome analysis.

Screening data from randomised participants who do not receive a second-line treatment in the ED will be collected and analysed in conjunction with randomised and treated participants.

**Fig. 2:**
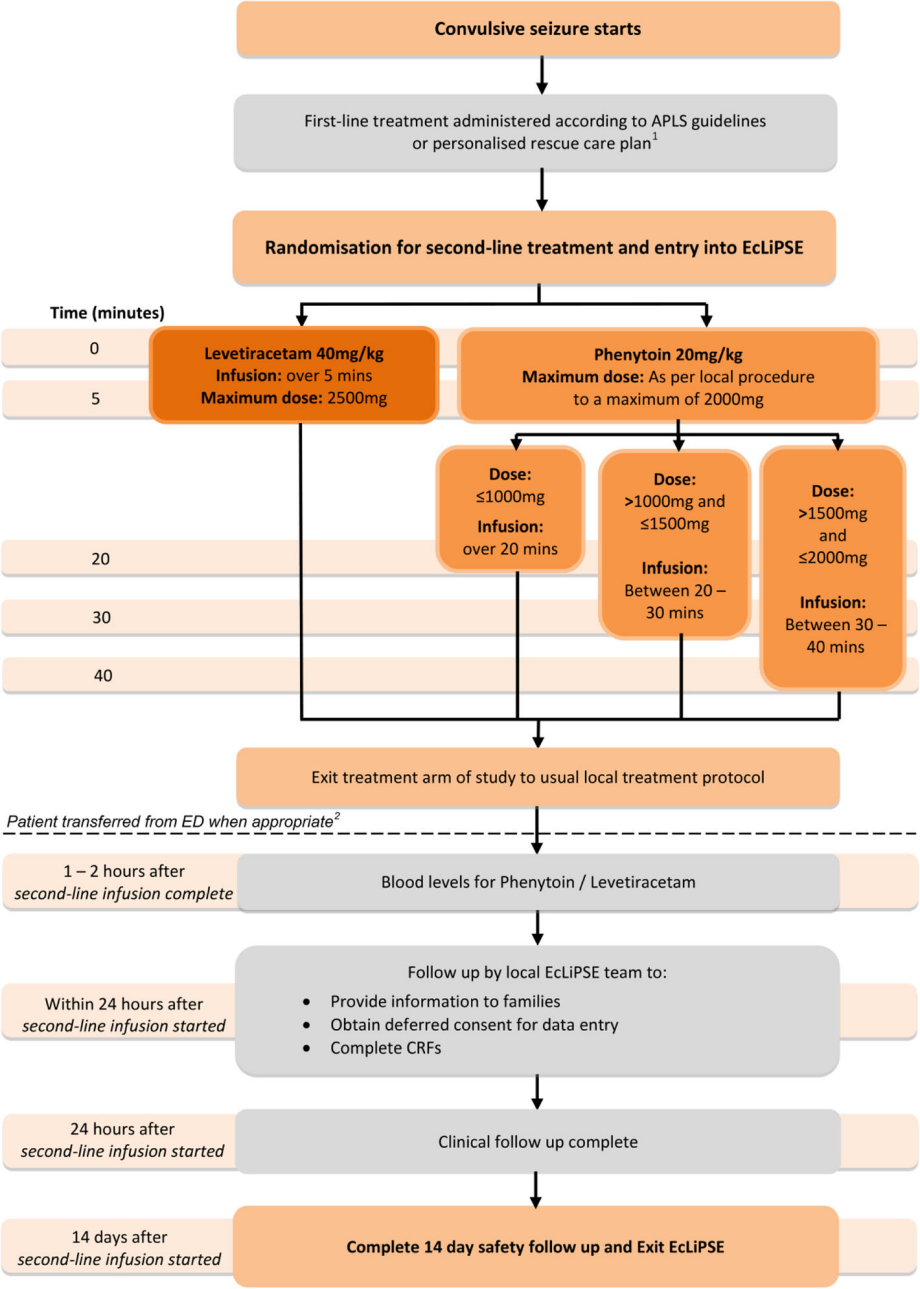
Schematic of Emergency treatment with Levetiracetam or Phenytoin in Status Epilepticus in children (EcLiPSE) study design. ^1^Administration of the first-line treatment may have occurred prior to arrival in the ED. ^2^If a patient is randomised but *not* treated with a second-line anticonvulsant, follow-up would end at this point. *APLS* advanced paediatric life support, *CRF* case report form, *ED* Emergency Department

Participants are randomised to levetiracetam or phenytoin in a ratio of 1:1 (Fig. 2), and the randomisation code list is generated by an independent statistician. Randomisation packs are sequentially numbered opaque tamperproof envelopes, to be opened in ascending order. Checks are performed periodically to ensure the correct number of randomisation packs is present, that they are intact, and that the sequential numbering system is maintained. The envelopes contain the first case report form (CRF) which is completed in the ED during the CSE episode. Data collected include time of drug administration, CSE cessation, additional therapy required, adverse events, and admission location.

### Follow-up

**Fig. 3:**
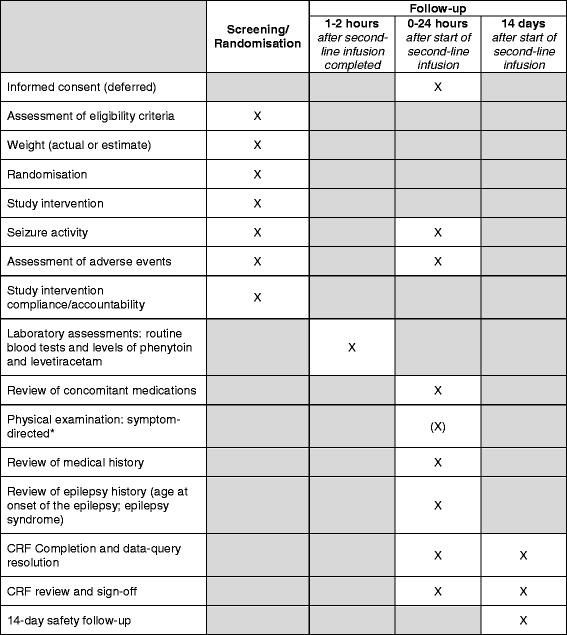
Emergency treatment with Levetiracetam or Phenytoin in Status Epilepticus in children (EcLiPSE) study assessments. *This physical examination should include a comment on any focal neurological signs. *CRF* case report form, *X* as indicated/appropriate

There are three time-periods for data collection in the EcLiPSE study (Figs. 1, 2, and 3). The first is in the ED during the acute CSE treatment phase. The second is the 24 h after allocated treatment, wherein data collected include further seizures, concomitant anticonvulsants that may have been required to treat other acute seizures, and adverse events. Finally, safety follow-up is undertaken 14 days after administration of the randomised treatment by review of hospital notes and a single-sheet, four-question questionnaire completed by the child’s parents. This includes information on whether there have been further admissions or organ failure.

### Blood samples

Samples are taken 1–2 h after completion of the randomised treatment to measure drug levels, a common practice when giving phenytoin [28]. Levetiracetam levels are measured in an accredited central laboratory, to which samples are not transferred until study consent has been obtained. Measurement of phenytoin levels is undertaken in the treating hospital.

### Trial treatments

EcLiPSE is an open-label trial using investigational medicinal products (IMPs) with marketing authorisation in the UK. These only become IMPs when the packaging is opened in the setting of this study. IMP provision is the responsibility of each site in accordance with standard clinical practice, and they are stored in line with local requirements for general medicine supplies.

A single dose of the randomly allocated treatment is administered by IV infusion. The levetiracetam dose is 40 mg/kg (maximum 2500 mg) over 5 min, diluted to a maximum of 50 mg/ml with 0.9% sodium chloride. The phenytoin dose is 20 mg/kg (maximum dose 2000 mg) at a rate not exceeding 1 mg/kg/min (or longer than 20 min for doses of >1 g), diluted with 0.9% sodium chloride to a maximum concentration of 10 mg/ml.

The allocated treatment is prepared and administered in accordance with standard clinical care with independent checking performed by two trained personnel. Trial-specific labelling is not required; rather, an approved “intravenous additive label” is used. If the randomised treatment is discontinued prior to administration of the full dose this is recorded. If CSE persists at the end of the IMP infusion, further medical management is decided by the local clinical team independent of the trial protocol (Fig. 2).

### Consent study

EcLiPSE includes a mixed method study (consent study) involving participants and trial recruiters to explore: 1) how information about the trial and deferred consent is exchanged during recruitment discussions; 2) views on deferred consent and participant decision making; 3) the impact of an un-blinded trial design; and 4) trial recruiter training [29–31]. The objective of the consent study is to identify potential barriers and solutions to recruitment and consent. This will inform recruiter training both during EcLiPSE and also inform approaches to recruitment and consent for future paediatric critical care trials. The consent study involves the following.

#### Audio-recording of recruitment and consent discussions between families and trial recruiters

EcLiPSE recruiters seek verbal permission to audio-record recruitment consultations when they first approach families about EcLiPSE; if permission is declined the recruitment consultation is not recorded. If there is more than one trial discussion, then all are recorded. Audio-recording occurs for the first 4 months of the trial at each site, or until data saturation is achieved [32].

#### Questionnaires completed by participant representatives after EcLiPSE consent discussions

EcLiPSE trial recruiters will invite all participants (including those who decline deferred consent) to complete an online or paper consent study questionnaire. If more than one participant is involved in the consent discussion, then all are invited to complete a questionnaire.

#### Telephone interviews with up to 25 parents of patients, 16- to 18-year-old patients with capacity, and one member of each site team

EcLiPSE recruiters ask participants who agree or decline consent if they wish to take part in a telephone interview. If they agree, the consent study researcher contacts them to arrange interviews within 1 month of the discussion. Interviews are likely to be conducted until data saturation is reached [32]. One member of each site team (Principal Investigator (PI) or research nurse) is emailed by the consent study researcher asking them to participate in a telephone interview within the first 4 months of site opening

#### Focus groups with EcLiPSE trial recruiters

At the end of the first year, the consent study researcher will invite EcLiPSE staff in 6–10 sites to participate in a focus group. Selection of sites for focus groups will be based on recruitment rates (both high and low) and recruitment issues identified in the analysis of audio-recordings, questionnaires, and parent interviews

Table 2 summarises when each section of the consent study is applicable to participants who have been randomised and administered treatment. Participants can select which study elements they wish to take part in during the consent process.

**Table 2:** Consent study applicability

Consent sought from	Location deferred consent sought	Applicable sections of the consent study		
		Audio-recording	Questionnaire	Interview
Parent/legal representative	On-site	✓	✓	✓
Bereaved parent/legal representative	On-site			✓
Adult with capacity	On-site	✓		✓
Parent/legal representative	Home		✓	✓
Bereaved parent/legal representative	Home			✓
Adult with capacity	Home			✓

### Statistical considerations

A separate and full statistical analysis plan will be developed prior to the final analysis of the trial and agreed by the Trial Steering Committee (TSC).

#### Sample size estimation

Sample size estimation is based on published seizure cessation rates for phenytoin (50–60%) [6] and levetiracetam (76–100%) [10–18, 22, 23]. A sample size of 140 participants in each group at a 0.05-level two-sided log-rank test for equality of survival curves will have 80% power to detect an increase in seizure cessation rates from 60% to 75%, (a constant hazard ratio of 0.661). A total of 308 randomised participants with deferred consent and given randomised treatment will allow for 10% loss to follow-up.

#### Statistical analysis plan

The primary analysis will be by ‘intention-to-treat’. A 5% level of statistical significance will be used throughout and all results will be presented with 95% confidence intervals. The primary outcome is a time to event outcome and will be analysed using the log-rank test and Kaplan-Meier curves. Dichotomous outcomes will be analysed using the chi-square test and presented with relative risks. Adjusted analyses will be conducted using Cox Proportional Hazards models or logistic regression, as appropriate. Variables included in the models will be determined from known prognostic factors. Adverse events will be presented using descriptive statistics. Reasons for missing data, and rates and reasons for not obtaining deferred consent, will be monitored.

#### Consent study analysis

Consent study data analysis will be assisted using the NVivo 8 qualitative data analysis package and SPSS software for statistical analysis. Quantitative analysis will involve descriptive statistics and the chi-square test for trend. Qualitative data will be analysed thematically [33]. Data from study methods will be analysed separately and then synthesised through the use of constant comparative analysis [34, 35].

### Safety monitoring

Safety is assessed by: 1) local research staff in the first 24 h after randomised treatment; 2) parent questionnaire and hospital record review 14 days after treatment administration; and 3) the Independent Data and Safety Monitoring Committee (IDSMC). Reporting procedures are determined by the nature of the event and investigators are provided with explanatory algorithms. All adverse events (AEs) up to 24 h after allocated treatment administration are recorded. Local investigators assign the severity of AEs as mild, moderate, or severe in line with definitions provided. A distinction is drawn between serious and severe AEs, and a severe AE need not necessarily be a serious AE (SAE). An AE whose causal relationship to the study drug is assessed as “possible”, “probable”, or “definite” is an adverse reaction (AR). Although ARs are not expected to occur more than 24 h following randomised treatment, investigators will report any AEs they feel are related, regardless of timing. Local investigators assign causality, though if there is doubt the Chief Investigator (CI) is notified.

On receipt of an SAE form, an assessment of expectedness is made by the CI. Serious unexpected events judged to be possibly, probably, or almost certainly related to the IMP are reported as suspected unexpected SARs (SUSARs). SARs, SAEs, and SUSARs are reported within 24 h of the local site becoming aware of the event, with additional information within 5 days if it is unresolved at the time of the initial report. Regulatory agencies, research ethics committees (RECs), PIs, and trial committees are notified of all SUSARs occurring during the study. All AEs are followed-up until the local PI deems there to be satisfactory resolution, the event is chronic, or the participant is stable.

Incorrect administration or overdose of IMP (20% or more above the recommended dose) is recorded. If this results in an AE or SAE, reporting occurs as previously described. Deaths during the reporting period are recorded as SAEs. The event or condition that caused or contributed to death is recorded, though if it is unknown it is recorded as “unexplained death”. If the cause of death subsequently becomes known, this is recorded.

### Trial monitoring

Quality control and quality assurance measures are in place to ensure that all elements of the EcLiPSE study are performed in compliance with applicable regulatory requirements. These are undertaken by the Trial Management Group (TMG), TSC, and IDSMC. An investigators’ meeting was hosted by the TMG, attended by PIs and site research nurses, to provide an overview of the EcLiPSE study. Site initiation visits are performed to deliver trial-specific training to key clinical and research personnel, which is subsequently cascaded to all relevant staff. Training and delegation logs are monitored for completeness, and appropriate approvals must be in place prior to site initiation. Screening, randomisation, and consent rates are monitored, and data are checked for consistency and missing or unusual values, with suspect data returned to the site as data queries to ensure their reliability and validity. The sponsor may also undertake site audits throughout the trial.

Independent oversight is provided by the IDSMC and independent members of the TSC. The IDSMC reviews and assesses recruitment, interim analysis of safety and effectiveness, trial conduct, and external data, and provides recommendations to the TSC concerning study continuation. The Haybittle-Peto approach will be employed for interim analyses with 99.9% confidence intervals but decisions around trial continuation will not be based on *p* values alone. The role of the TSC is to provide overall supervision for the trial and provide advice through its independent Chairperson. The ultimate decision for the continuation of the trial lies with the TSC.

### Confidentiality

Participant medical information is confidential, and disclosure to third parties is prohibited with the exception of name data which is transferred on informed consent/assent forms. This transfer of identifiable data is disclosed in the participant information sheet.

### Risk assessment

A risk assessment of potential patient, organisational, and study hazards was performed prior to commencement, involving all relevant stakeholders. The risks associated with this study are deemed to be ‘no higher than that of standard medical care’. This level of risk informs the regulatory requirements, nature, and extent of the monitoring, and the management processes used.

### Regulatory approval

This trial falls within the remit of the UK Statutory Instrument 2004 No 1031: Medicines for Human Use (Clinical Trials) Regulations 2004 as amended. This trial has been registered on EudraCT. The EudraCT reference is 2014-002188-13.

### Dissemination

The results of the EcLiPSE study will be published in peer-reviewed journals and in a report published by the National Institute for Health Research Health Technology Assessment programme (NIHR HTA). The TMG forms the basis of the Writing Committee which will follow the Uniform Requirements for Manuscripts Submitted to Biomedical Journals (http://www.icmje.org/). Publications will be distributed to participating centres, and throughout relevant networks and bodies. Families that consent to receiving a copy of the findings will be sent a lay summary. Findings will also be presented at relevant national and international scientific (e.g. epilepsy, emergency medicine, paediatric) conferences, and at meetings of relevant charities, accompanied by press releases and dissemination via other outlets.

## Discussion

This study has a number of opportunities and challenges which might affect recruitment. It is an example of paediatric research in an emergency situation and consequently has to involve either waived or deferred consent; waived consent is ethically unacceptable in this setting and research without prior consent will be a new concept to many participating centres. However, deferred consent was not an issue in a previous paediatric emergency research study, the results of which led to a change in national policy and practice in the management of paediatric CSE [36]. Early input and advice was obtained through patient and public involvement and all site training includes a video scenario of a professional mock interview in which the study is discussed and deferred consent is explained, processes designed to maximise recruitment into EcLiPSE. Early engagement with PERUKI optimised success through collaboration with clinicians and researchers in the development and delivery of the study, together with the selection of the most appropriate sites in which to recruit patients.

## Trial status

Recruitment to this study commenced in July 2015, and to date 160 patients have been enrolled. Twenty-nine sites are currently open to recruitment, with a plan to recruit patients at 30 sites in total across the UK and PERUKI network. Recruitment is scheduled to finish in March 2018 and analysis be completed by December 2018.

## Additional file

Additional file 1: SPIRIT 2013 checklist: recommended items to address in a clinical trial protocol and related documents. (DOCX 60 kb)

## Abbreviations

AE: Adverse event
APLS: Advanced paediatric life support
AR: Adverse reaction
CI: Chief Investigator
CRF: Case report form
CSE: Convulsive status epilepticus
EcLiPSE: Emergency treatment with Levetiracetam or Phenytoin in Status Epilepticus in children
ED: Emergency Department
IDSMC: Independent Data and Safety Monitoring Committee
IMP: Investigational medicinal product
IV: Intravenous
NIHR HTA: National Institute for Health Research Health Technology Assessment programme
PERUKI: Paediatric Emergency Research in the United Kingdom & Ireland
PI: Principal Investigator
PICU: Paediatric intensive care unit
REC: Research ethics committee
RSI: Rapid sequence intubation
SAE: Serious adverse event
SAR: Serious adverse reaction
SUSAR: Suspected unexpected serious adverse reaction
TMG: Trial Management Group
TSC: Trial Steering Committee
